# Simultaneous inhibition of JAK and SYK kinases ameliorates chronic and destructive arthritis in mice

**DOI:** 10.1186/s13075-015-0866-0

**Published:** 2015-12-10

**Authors:** Alba Llop-Guevara, Mónica Porras, Carla Cendón, Irene Di Ceglie, Francesco Siracusa, Federica Madarena, Vagelis Rinotas, Lluís Gómez, Peter L. van Lent, Eleni Douni, Hyun Dong Chang, Thomas Kamradt, Juan Román

**Affiliations:** Draconis Pharma S.L., Calle Pallars 179, Barcelona, Spain; Deutsches Rheuma-Forschungszentrum, Berlin, Germany; Radboud University Medical Center, Nijmegen, Netherlands; Universitätsklinikum, Jena, Germany; Laboratory of Genetics, Department of Biotechnology, Agricultural University of Athens, Athens, Greece; Biomedical Sciences Research Center “Alexander Fleming”, Vari, Greece

**Keywords:** Arthritis, JAK, SYK, Small molecule inhibitors, Anti-TNF therapy, Fibroblast-like synoviocytes, Osteoclasts, Memory cells

## Abstract

**Introduction:**

Despite the broad spectrum of antirheumatic drugs, RA is still not well controlled in up to 30-50 % of patients. Inhibition of JAK kinases by means of the pan-JAK inhibitor tofacitinib has demonstrated to be effective even in difficult-to-treat patients. Here, we discuss whether the efficacy of JAK inhibition can be improved by simultaneously inhibiting SYK kinase, since both kinases mediate complementary and non-redundant pathways in RA.

**Methods:**

Efficacy of dual JAK + SYK inhibition with selective small molecule inhibitors was evaluated in chronic G6PI-induced arthritis, a non-self-remitting and destructive arthritis model in mice. Clinical and histopathological scores, as well as cytokine and anti-G6PI antibody production were assessed in both preventive and curative protocols. Potential immunotoxicity was also evaluated in G6PI-induced arthritis and in a 28-day TDAR model, by analysing the effects of JAK + SYK inhibition on hematological parameters, lymphoid organs, leukocyte subsets and cell function.

**Results:**

Simultaneous JAK + SYK inhibition completely prevented mice from developing arthritis. This therapeutic strategy was also very effective in ameliorating already established arthritis. Dual kinase inhibition immediately resulted in greatly decreased clinical and histopathological scores and led to disease remission in over 70 % of the animals. In contrast, single JAK inhibition and anti-TNF therapy (etanercept) were able to stop disease progression but not to revert it. Dual kinase inhibition decreased Treg and NK cell counts to the same extent as single JAK inhibition but overall cytotoxicity remained intact. Interestingly, treatment discontinuation rapidly reversed such immune cell reduction without compromising clinical efficacy, suggesting long-lasting curative effects. Dual kinase inhibition reduced the Th1/Th17 cytokine cascade and the differentiation and function of joint cells, in particular osteoclasts and fibroblast-like synoviocytes.

**Conclusions:**

Concurrent JAK + SYK inhibition resulted in higher efficacy than single kinase inhibition and TNF blockade in a chronic and severe arthritis model. Thus, blockade of multiple immune signals with dual JAK + SYK inhibition represents a reasonable therapeutic strategy for RA, in particular in patients with inadequate responses to current treatments. Our data supports the multiplicity of events underlying this heterogeneous and complex disease.

**Electronic supplementary material:**

The online version of this article (doi:10.1186/s13075-015-0866-0) contains supplementary material, which is available to authorized users.

## Introduction

Many patients with rheumatoid arthritis (RA) continue with persistently active disease. The conventional disease-modifying anti-rheumatic drugs (DMARDs), most commonly methotrexate (MTX), non-steroidal anti-inflammatory drugs (NSAIDs) and steroids, can inhibit local inflammatory symptoms but have little or insufficient long-lasting systemic effects. This prompted the emergence of biologic agents targeting specific intercellular signals, such as antagonists of TNF, IL-6 and IL-1β, which apart from their anti-inflammatory effects directly reduce the pathogenic activity of synovial cells. Biologic agents targeting specific immune cells, such as modulators of B cell and T cell activity, were approved for RA patients not responding adequately to oral DMARDs or other biologic agents. While biologic agents have greatly improved the effectiveness of RA treatment, 30–40 % of patients still do not have an appropriate response and, therefore, have interruptions in treatment [1]. Indeed, their required chronic use increases the risk of developing cancer and infection, and can cause drug resistance. In addition, biologic agents are expensive, have long half-lives and must be injected or infused, which highlights the need for better therapies.

Small molecule drugs, such as inhibitors of protein kinases, appear to be a good alternative due to their ability to immunomodulate multiple key intracellular signals. Furthermore, these chemical compounds (<1 kDa) are orally available and have a short half-life, which facilitates treatment modulation. Persuasive preclinical data support the targeting of specific members of the mitogen-activated protein kinase (MAPK) (e.g., p38-α) and PI3K/Akt/mTOR (p110δ) pathways but none of the inhibitors have progressed to phase III clinical studies due to lack of efficacy in RA patients and concerns about toxicity (reviewed in [2, 3]). The focus is now placed on kinases higher in the signaling cascade, such as the non-receptor cytoplasmic tyrosine kinases Janus kinase (JAK) and the spleen tyrosine kinase (SYK).

The JAK family in mammals includes the closely related isoforms JAK1, JAK2, JAK3 and TYK2 (tyrosine kinase 2), which homo/heterodimerize and bind to cytokine receptors. The JAK/STAT signaling pathways mediate the effects of many cytokines/interferons and growth factors important in RA (e.g., IL-2, IL-6, IL-7, IL-10, IL-12, IL-15, IL-21, IL-23, interferons (IFNs), granulocyte macrophage colony-stimulating factor (GM-CSF)) and regulate the activity of hemopoietic and joint resident cells. In fact, the relevance of some of these signals has been validated in the clinics through their blockade using specific biologic drugs [4, 5]. It is, thus, not surprising that JAK inhibitors have proved efficacious in animal models of arthritis [6, 7], and in clinics [8, 9]. Indeed, the small pan-JAK inhibitor tofacitinib (CP-690,550) was approved for the treatment of moderate to severe RA in patients who do not respond to MTX or conventional synthetic and biological DMARDs [10, 11]. JAK inhibition has demonstrated high efficacy, as approximately 60–70 % RA patients experience clinical improvement with at least 20 % response according to American College of Rheumatology criteria (ACR20 response) [12], even in non-responders to anti-TNF treatment (ACR20 of 48 %) [13]. As such, Lee et al*.* recently suggested that tofacitinib could be used as first-line monotherapy for RA [14].

SYK kinase is required for the signal transduction of receptors that associate with transmembrane proteins containing immunoreceptor tyrosine activation motifs (ITAM), i.e., the B cell receptor, T cell receptor and certain Fc receptors primarily present in granulocytes, dendritic cells (DCs) and macrophages. SYK additionally mediates signaling by integrins and members of the lectin/selectin families [15] and is involved in the activity of non-immune cells, such as fibroblast-like synoviocytes (FLS) and vascular endothelial cells [16–18]. As SYK is implicated in several pathways linked to RA pathogenesis, SYK inhibition is viewed as a plausible therapeutic strategy. To our knowledge, selective SYK inhibitors, such as PRT062607 (Portola/Biogen Idec), have shown encouraging preclinical data [19] but their potential efficacy in RA patients has not been evaluated. Here, we investigated whether the high efficacy of JAK inhibition could be improved by concurrently inhibiting SYK. To this end, we used potent and selective small molecule inhibitors of pan-JAKs (tofacitinib) and SYK (PRT062607) either in combination or alone, which were tested, for the first time, in a destructive and non-remitting arthritis murine model [20, 21].

## Methods

### Induction and scoring of arthritis

DBA/1 mice (six w.o. males from Janvier, France) were immunized subcutaneously (s.c.) (100 μl at each side of the base of the tail) with 400 μg recombinant human (rhu) glucose-6-phosphate isomerase (G6PI) emulsified in complete Freund’s adjuvant (CFA, Sigma-Aldrich, St. Louis, MO, USA) on day 0, as previously described [20]. The indicated amount of antigen was mixed with CFA in a 1:1 ratio (v/v) and emulsified with a Polytron. When specified, regulatory T cells (Tregs) were depleted injecting intraperitoneally (i.p.) 400 μg anti-CD25 Ab (PC61.5, BioXcell, West Lebanon, NH, USA) 11 and 8 days before immunization [21].

The mouse weight was recorded and the clinical score was evaluated over time. Each paw section was scored separately, and these scores were all added together as follows:

Total score per mouse = (Sum of scores of 2 wrists + 2 ankles (i.e., max 12)) + (Sum of scores of 2 metacarpals + 2 feet (i.e., max 12)) + (Number of inflamed fingers (max 8) + toes (max 10)/2 (i.e., max score of 9)).

For each paw section, a score of 0 to 3 was assigned, where 0 indicates no clinical signs of arthritis (healthy state), 1 and 2 indicate mild/intermediate swelling and redness of the paw, and 3 indicates massive swelling, redness and burst skin.

All experiments with mice were approved by the Animal Experimentation Ethical Committee of Draconis Pharma, the Animal Experimentation Commission of the Generalitat de Catalunya (Catalonian Government) or the German federal state institution Landesamt für Gesundheit und Soziales*.*

### T cell-dependent antibody response (TDAR) model

CD-1 mice (six to eight w.o. females from Janvier, France) were immunized on day 0 with 300 μg of keyhole limpet hemocyanin (KLH, Sigma-Aldrich, subplantar injection of 30 μl). On day 14, mice were sacrificed and blood, plasma and spleens were obtained.

### Treatments

The kinase inhibitors Tofacitinib (JAKi) and PRT-062607 (SYKi) were synthesized by Aptonchem Co. Ltd. (Hangzhou, China) or in house (Draconis Pharma), respectively. These compounds were diluted in water, sonicated for 5 minutes and given orally (p.o.) (20 mg/kg JAKi and 30 mg/kg SYKi) once daily (QD) starting on day 0 or 12, unless otherwise specified. We corrected the amount of each compound used by the molecular weight (MW) of the free amine: MW of JAKi = 504.49 g/mol (free amine 312.37 g/mol) and MW of SYKi = 453.50 g/mol (free amine 393.45 g/mol). Prednisolone (Sigma-Aldrich, 3 mg/kg) was also diluted in water and given p.o. QD. The soluble dimeric human TNFR p75–IgG-Fc fusion protein (etanercept, Enbrel®, Pfizer, Thousand Oaks, CA, USA) was diluted in PBS and given s.c. (10 mg/kg) every third day.

### Sample collection

Samples were obtained at the indicated time points. Inguinal lymph nodes and spleens were weighted and single cell suspensions were made in Roswell Park Memorial Institute (RPMI)1640 medium (Life technologies, ThermoFisher Scientific, Eugene, OR, USA, supplemented with 10 % FBS, glutamine, 100 U/ml penicillin, 100 mg/ml streptomycin) by mechanical disruption of the tissue with a syringe plunger on a 40-μm cell strainer. Peripheral blood was collected in tubes with 0.5 % K_2_-EDTA, hematological parameters analyzed by Celltac E analyzer (Nihon Khoden, Tokyo, Japan) and plasma stored at −80 °C for cytokine and Ig quantification. Ankle swelling was measured using a dial-gauge caliper (Peacok, Ozaki MFG Co. Ltd., Tokyo, Japan); the average thickness of both hind legs was used. The right hind limb of each mouse was fixed in 10 % formalin and then decalcified with Osteomoll (Merck Millipore) for histopathological assessment.

### Cytokine production by splenocytes

Splenocytes were plated in triplicates (8 × 10^5^ cells/well in 96-well flat-bottom plates) and re-stimulated with either 20 μg/ml rhu G6PI or PBS in RPMI medium supplemented with 10 % FBS, 100 U/ml penicillin and 100 μg/ml streptomycin at 37 °C in 5 % CO_2_ for 72 h. When indicated, splenocytes from non-treated arthritic mice were also exposed to 0.5 μM of JAKi and 0.5 μM of SYKi for 72 h. Supernatants were collected and murine IFNγ, IL-17A and IL-6 were measured by ELISA (BD, Franklin Lakes, NJ, USA,  and Affymetrix, Santa Clara, CA, USA and eBioscience, San Diego, CA, USA), according to manufacturer’s protocols.

### G6PI- and KLH-specific Ig ELISA

Plasma levels of G6PI- and KLH-specific antibodies were measured by ELISA. Briefly, 96-well flat-bottom plates were coated with 5 μg/ml of G6PI or 10 μg/ml of KLH in carbonate buffer (0.1 M Na_2_CO_3_ and NaHCO_3_, pH 9.5) overnight at 4 °C, washed with PBS containing 0.05 % Tween-20 (Sigma-Aldrich) and incubated with a blocking buffer (2 % BSA (Sigma-Aldrich) in PBS) for 1 h to saturate non-specific binding sites. Serial 5-fold or 10-fold dilutions of the plasma in PBS + 0.05 % Tween + 2 % BSA were added for 2 h followed by 1 h incubation with isotype-specific Ab with peroxidase (goat anti-mouse Fcγ- or Fcμ-specific Ab-HRP, Jackson ImmunoResearch, Suffolk, UK). After washing, the substrate 3,3′-5,5′ tetramethylbenzidin (TMB) (BD) was added, the reaction was stopped with 1 M H_2_SO_4_ and the absorbance was determined at 450 nm with a reference wavelength of 570 nm.

### Immunophenotyping by flow cytometry

Cell surface phenotyping was performed by using anti-mouse antibodies to CD3, CD4, CD8, CD19, CD11c, CD11b, MHCII, CD86, CD49b, CD25, CD44, CD138 and/or fixable live/dead. For intracellular markers, cells were fixed, permeabilized and stained with anti-Foxp3, CD40L, IFNγ, IL-2, TNFα, 5-Bromo-2′-deoxyuridine (BrdU), ovalbumin (OVA), κ and/or λ. Cells were analyzed with a FACS Calibur or a LSR Fortessa flow cytometer (BD Biosciences) and data were analyzed using FlowJo software.

### Cell-mediated cytotoxicity (CDC) assay

Murine splenocytes (effector cells, EC) were isolated from treated and untreated mice. Target cells (TC, YAC-1 cell line) were labeled with 2.5 μM CFSE fluorescent dye (Life technologies) to separate them from the EC population. Untreated splenocytes were treated in vitro for 1 h at 37 °C with JAKi + SYKi or anti-TNF, as indicated. Then, labeled TC were co-incubated with EC (ratio of 1 TC:10 EC) for 4 h and the dye PI (Life technologies) was added to discriminate live and dead cells.

### Phagocytosis assay

Peripheral blood mononuclear cells (PBMCs) were isolated from human buffy coats by Ficoll density gradient. Cells (2 × 10^6^ cells/tube) were pre-treated with JAKi and/or SYKi for 1 h. Media were removed and replaced with 1 mg/ml Red pHrodo^TM^ BioParticles® suspension (Life Technologies) and incubated for 1.5 h at 37 °C. Phagocytosis of particles was quantified by flow cytometry.

### Gene expression

Total RNA was purified from splenocytes using the PureLink RNA Mini kit (Ambion, ThermoFisher Scientific, Carlsbad, CA, USA) and the concentration and purity were assessed using a Safire spectrophotometer (Tecan, Männedorf, Switzerland). RNA (1 μg) was retro-transcribed to cDNA using High-Capacity cDNA Reverse Transcription kit (Life technologies) following manufacturer’s instructions. RANKL, IFNγ and IL-6 expression were evaluated by quantitative real-time PCR using TaqMan Universal Master Mix and a 7500 Real Time PCR System (Applied Biosystems, ThermoFisher Scientific). Relative quantification was determined using the comparative method: 18S ribosomal RNA was used as the housekeeping gene (Mm04277571_s1). Taqman probes for RANKL, IFNγ and IL6 were Mm00441906_m1, Mm00801778_m1 and Mm00446190_m1, respectively (Applied Biosystems).

### Histopathological assessment

Tissue samples were paraffin-embedded and longitudinal microsections were stained with H&E (Merck Millipore), according to standard procedures. A score of 0 (normal) to 5 (strongly affected) was given based on the degree of inflammation, bone erosion, cartilage damage and pannus formation in tarsus and phalanges, as previously described [22, 23]. Experienced pathologists at AnaPath (Spain) processed the tissues and performed the blinded histopathological assessment.

### Fibroblast-like synoviocyte (FLS) isolation, stimulation and invasion assay

Small joints from Treg-depleted mice at day 56 post-immunization were dissected and digested in 1 mg/ml collagenase type IV solution for 2 h (Worthington Biochemical Corp., Lakewood, NJ, USA 250 U/mg). FLS were cultured in DMEM (Sigma-Aldrich) supplemented with 10 % FBS, 100 U/ml Penicillin/Streptomycin (Jena Bioscience, Jena, Germany) and 10 mM Hepes (Serva, Heidelberg, Germany). All the FLS used were between passages 4 and 5. FLS were plated (5 × 10^4^ cells/well in 24-well plates) and stimulated with 10 ng/ml rmTNF-α and 50 ng/ml of rmIL-17A (both Peprotech, London, UK) or left unstimulated. All cells were also treated with 0.5 μM JAKi and/or SYKi; dimethyl sulfoxide (DMSO) only was used as a control. After 24 h of incubation, the supernatants were harvested and IL-6, matrix metalloproteinase-3 (MMP-3) and MMP-9 were quantified by ELISA (reagents from eBioscience, San Diego, CA, USA and Peprotech for IL-6; commercial DuoSet kits from R&D Systems, Minneapolis, MN, USA for MMPs).

To test FLS invasiveness, a transwell system was used. ThinCerts™ inserts with 8-μm pores (Greiner bio-one, Frickenhausen, Germany) were coated with a bovine collagen solution (PureCol ®, Advanced BioMatrix, Carlsbad, CA, USA; 10 × MEM, Gibco, ThermoFisher Scientific, Waltham, MA, USA; sodium bicarbonate 7.5 %, Gibco) and put in 24-well plates. FLS (2 × 10^4^ cells/well) resuspended in culture medium without FBS in the presence of 0.5 μM of JAKi and/or SYKi or DMSO only, were seeded on top of the collagen matrix. Culture medium with 10 % FBS was used as chemoattractant. After 48 h of incubation, the cells that did not migrate were removed from the upper part of the insert with a cotton swab and the lower part of the insert was stained with crystal violet. The percentage of invaded area was calculated using the program Fiji, as described [24].

### Osteoclast differentiation and bone resorption assay

Bone marrow cells were collected after flushing out of femurs and tibiae, subjected to gradient purification using ficoll-paque (GE Healthcare, Little Chalfont, Buckinghamshire, UK), plated in 96-well plates at a density of 6 × 10^4^ cells/well and cultured in αMEM medium (Gibco) containing 10 % FBS supplemented with 40 ng/ml rank ligand (RANKL) (R&D Systems) and 25 ng/ml M-CSF (R&D Systems) [25] in the presence or absence of CP and PRT compounds for 5 days. To visualize osteoclasts, cell cultures were stained with tartrate-resistant acid phosphatase (TRAP), using an acid phosphatase leukocyte (TRAP) kit (Sigma-Aldrich).

To assess osteoclast activity, bone marrow cells were seeded on 650-μm-thick bovine cortical bone slices (10^5^ cells/slice) in αMEM supplemented with 5 % FBS, 20 ng/ml RANK-L and M-CSF (30 ng/ml). Medium was refreshed after 3 days. Differentiated osteoclasts were treated with DMSO or 0.5 μM of JAKi and/or SYKi on day 5 and bone resorption was assessed 48 h later. Osteoclasts were lysed with water and mechanically removed by sonicating the bone slices in 10 % NH_3_ for 20 minutes. Finally, bone slices were washed, incubated for 10 minutes with 10 % hydrated potassium aluminium sulfate, washed again and stained with Coomassie blue (PhastGel Blue R-350; GE Healthcare Bioscience, Uppsale, Sweden) in order to visualize the pits of resorption. Five micrographs per slice were acquired with a digital camera mounted on an inverted light microscope and the percentage of eroded area was quantified using the program Leica Application Suite (version 4.3.0).

### Memory cell

BALB/c mice (Charles River, Germany) were immunized i.p. with 100 μg OVA (Sigma-Aldrich) in 100 μl alum (ThermoFisher Scientific) at day 0. On day 21, mice were boosted with 100 μg OVA in 100 μl alum. From day 20 to 35, mice received 1 mg/ml BrdU (Sigma-Aldrich) dissolved in drinking water to label newly generated plasma cells (i.e*.*, plasma cells generated after boost). From day 35 to 46, mice were treated with 20 mg/kg CP and/or 30 mg/kg PRT (p.o. QD). On day 46, single cell suspensions were generated from spleen and bone marrow and stained for flow cytometry. For the ex vivo re-stimulation assay, cells were plated in complete RPMI medium in 12-well plates (10^7^ cells/well) and re-stimulated for 6 h with 100 μg OVA. Brefeldin A (BioLegend, London, UK) was added after the first 4 h and finally stained for flow cytometry.

### Statistical analysis

Data are expressed as mean ± standard error of the mean and were analyzed and graphed with Prism software version 5 (GraphPad, La Jolla, CA, USA). Statistical analysis was calculated using Student’s *t* test (unpaired, two-tailed) and one-way or two-way analysis of variance (with the Dunnett or Bonferroni post hoc test). Differences were considered statistically significant when the *p* value was *<*0.05.

## Results

### Dose selection of potent and selective small JAK and SYK inhibitors

We used tofacitinib (CP-690,550, designed by Pfizer) as a selective pan-JAK inhibitor (JAKi) and PRT062607 (Portola Pharmaceuticals Inc. and Biogen Idec Inc.) to specifically inhibit SYK (SYKi), or their combination (JAKi + SYKi). In Additional file 1, we show data obtained to determine the doses of inhibitors to be further used for in vivo studies. Briefly, we first confirmed the potency and selectivity of these inhibitors in biochemical and cellular assays (half maximal inhibitory concentration (IC_50_) (JAK) approximately 0.02 μM and IC_50_ (SYK) approximately 0.5 μM). We also determined their efficacy in standard acute arthritis models in mice; namely the collagen-induced arthritis (CIA) model, which strongly relies on T cell function and therefore eases the study of JAK inhibition (ED_50_ (JAK) approximately 11 mg/kg QD), and the collagen antibody-induced arthritis (CAIA) model for SYK inhibition, as it requires processing of anti-type II collagen antibodies and immune complexes via FcR. Based on these results, we decided to use a dosage of 20 mg/kg of tofacitinib (JAKi) and 30 mg/kg of PRT062607 (SYKi) QD. A single oral dose of tofacitinib or PRT062607 in naive mice resulted in a rapid increase of plasma levels (t_max_ = 1–3 h), reaching maximal concentrations that did not compromise kinase selectivity, and were completely cleared daily. Therefore, the selected doses guaranteed high efficacy while maintaining selectivity.

### Dual JAK + SYK inhibition prevents G6PI-induced arthritis

To test the efficacy of dual JAK + SYK inhibition, we chose a novel and well-accepted murine model of arthritis induced by the systemic antigen G6PI. Immunization is achieved by administering G6PI along with CFA subcutaneously. The resulting acute, self-limited type of arthritis [20] can be switched deliberately to a non-self-remitting and destructive type by transiently depleting Treg cells prior to disease induction [21]. This chronic model is characterized by exacerbated clinical signs of inflammation (redness and paw swelling), joint deformations and higher levels of T helper (Th1) and Th17 cytokines compared to the acute type (see [21] and Additional file 2). Here, we were interested in mimicking severe and non-self-remitting disease, as it better represents human disease; therefore, we used the chronic G6PI model.

**Fig. 1:**
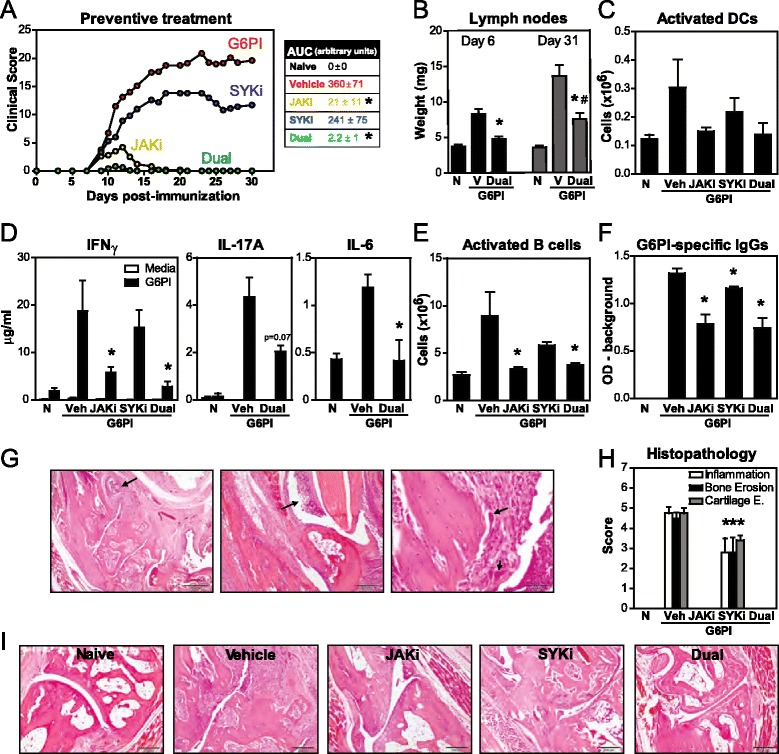
Efficacy of Janus kinase (*JAK*) + spleen tyrosine kinase (*SYK*) inhibition as a preventive treatment in glucose-6-phosphate isomerase (*G6PI*)-induced arthritis. DBA/1 mice were immunized with G6PI + complete Freund’s adjuvant on day 0, after T regulatory cell depletion with anti-CD25 Ab (days −8 and −11). Treatments (20 mg/kg of tofacitinib - JAK inhibitor (*JAKi*) - and/or 30 mg/kg of PRT062607 – SYK inhibitor (*SYKi)*) were orally administered daily from day 0. Samples were obtained on day 31 unless otherwise specified. **a** Time course for arthritis scores and resulting areas under these score curves. **b** Mean weight of both inguinal lymph nodes at day 6 and day 31 post-immunization. **c** Numbers of CD11c^hi^MHCII^+^CD86^+^ activated dendritic cells (DCs) in the spleen. **d** Cytokine secretion by splenocytes stimulated in vitro with 20 μg/ml recomninant human G6PI for 72 h. **e** Numbers of CD19^+^MHCII^+^CD86^+^ activated B cells in the spleen. **f** Levels of G6PI-specific IgGs in plasma. **g** Histological features of G6PI-induced arthritis, including severe inflammation and bone/cartilage destruction of tarsal and phalangeal joints from hind paws (H&E staining). *Arrows* cell aggregates (*left*, *bar* 500 μM), infiltration of lymphocytes, macrophages and neutrophils in the synovium (*middle*, *bar* 200 μM) and osteoclasts (*right*, *bar* 50 μM). **h** and **i** Data and representative histological sections of tarsal joints (*bar* 500 μM). Graphs show mean (+ standard error of the mean) for 3–5 mice/group, *p <0.05 versus vehicle control, ^#^ *p* <0.05 versus non-immunized and untreated group (naive). *N* naive, *V/Veh* vehicle, *IFN* interferon

In order to study the impact of kinase inhibition on arthritis development, daily oral treatment with JAKi and/or SYKi was started upon immunization (see a diagram of this experimental protocol in Additional file 2). Figure 1a shows that mice receiving SYKi developed moderate and persistent arthritis compared to vehicle controls and ended with 40 % lower clinical scores. Mice treated with JAKi had mild inflammation of the paws, except for the toes and fingers, for a week only, as clinical signs were resolved in all mice after 17 days of treatment. As a measure for the aggregate effect of these treatments over time, the average area under the clinical score curve decreased by 94 % with JAKi, but by only 33 % with SYKi. The greatest effects were observed with the dual inhibition of JAK + SYK, which prevented all mice from developing arthritis. None of the mice had inflammation of the toes/fingers and only 30 % were scored with incipient swelling of the wrists/ankles for at most 4 days. Accordingly, lymph node weights were significantly decreased compared to the progressively bigger ones in arthritic controls over time (Fig. 1b). In the spleen, the CD11c^hi^ MHCII^+^ dendritic cell pool (not shown), and consequently the number of activated (CD86^+^) DCs (Fig. 1c), were reduced in mice treated with JAKi + SYKi, likely contributing to minimize antigen-specific immune responses.

Parameters of T and B cell activity support a critical role for JAK signaling in the induction phase of the disease. Splenocytes from treated mice were stimulated in vitro with G6PI for 72 h and cytokine production was quantified by ELISA. Levels of IFNγ, IL-17A and IL-6 were comparable in vehicle controls and SYKi-treated mice (Fig. 1d and not shown). In contrast, JAKi decreased cytokine production and a greater reduction was observed upon dual JAKi + SYKi, confirming milder Th1/Th17 responses in these groups. Similarly, CD86^+^-activated B cells and levels of G6PI-specific IgGs were significantly lower in mice receiving JAKi or JAKi + SYKi (Fig. 1e and f).

A detailed histopathological evaluation of the hind limbs of arthritic mice revealed severe lesions in both tarsal (Fig. 1g-i) and phalangeal (not shown) joints with abundant immune and inflammatory cells (primarily lymphocytes, macrophages and neutrophils), pannus formation with neovascularization, marked cartilage and bone erosion, loss of trabeculae and presence of more fibroblasts and osteoclasts. Dual JAK + SYK inhibition prevented paw inflammation and bone/cartilage damage. Most of these effects were driven by JAK inhibition, which completely cleared an initial mild inflammatory response and prevented bone and cartilage erosion. With SYKi the histopathology score was reduced by 30-40 % at day 31.

### Dual JAK + SYK inhibition ameliorates chronic and severe G6PI-induced arthritis

**Fig. 2:**
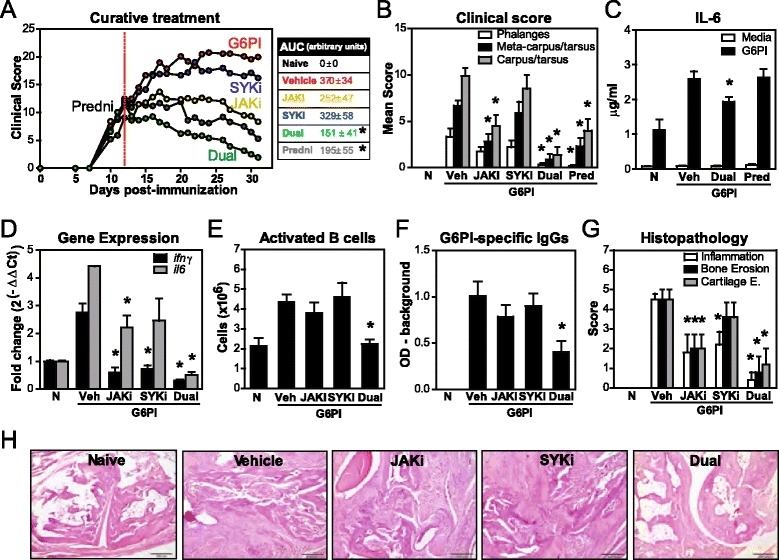
Efficacy of Janus kinase (*JAK*) + spleen tyrosine kinase (*SYK*) inhibition as a curative treatment in glucose-6-phosphate isomerase (*G6PI*)-induced arthritis. DBA/1 mice were immunized with G6PI + complete Freund’s adjuvant on day 0, after T regulatory cell depletion with anti-CD25 Ab (days −8 and −11). Treatments were orally administered daily from day 12 (*red dotted line*). Samples were obtained on day 31. **a** Time course for arthritis scores and resulting areas under these score curves (*AUC*). **b** Detailed clinical score by paw segment at day 31. **c** Cytokine secretion by splenocytes stimulated in vitro with 20 μg/ml recombinant human G6PI for 72 h. **d** Cytokine expression in splenocytes. **e** Numbers of CD19^+^MHCII^+^CD86^+^ activated B cells in spleens. **f** Levels of G6PI-specific IgGs in plasma. **g** and **h** Data and representative histological sections of tarsal joints (*bar* 200 μM). Graphs show mean (+ standard error of the mean) for 3–7 mice/group, *p <0.05 versus vehicle control. *N* naive, *V/Veh* vehicle, *Pred/Predni* prednisolone

We next evaluated the therapeutic potential of dual JAK + SYK inhibition in a curative protocol, which is a more strenuous measure of activity and is clinically more relevant. In this case, oral daily treatment was started at day 12 post-immunization, when clinical signs, primarily inflammation in proximal and distal joints of paws, were present. The clinical score of vehicle control mice progressively increased, becoming stable around day 20 (Fig. 2a). Inhibition of JAK, but not SYK, was able to stop disease progression, and after 2 weeks of treatment started to mildly reduce arthritis severity. By day 31, mice treated with JAKi or prednisolone (3 mg/kg QD), a corticosteroid widely used as anti-inflammatory agent, had a reduction in overall clinical score of 58 % and 74 %, respectively. In contrast, dual JAKi + SYKi immediately prompted a gradual decrease in the clinical score for all joints (Fig. 2b) from all mice and over 70 % of these mice were totally cured (score of 0) after 3 weeks of treatment.

Upon in vitro G6PI stimulation of splenocytes from these mice, IL-6 production was only significantly reduced in the dual treatment group (Fig. 2c), confirming attenuation on antigen-specific responses. Th1 (IFNγ) and Th17 (IL-17A and IL-6) cytokine expression was also decreased (Fig. 2d and not shown). Furthermore, combined JAKi + SYKi was more effective at limiting B cell activation and function, as assessed by the levels of serum G6PI-specific IgG, relative to either inhibitor alone (Fig. 2e and f).

In terms of histopathological changes in tarsal joints (Fig. 2g and h), single JAKi or SYKi decreased around 50 % of joint inflammation, while dual treatment induced a reduction of over 90 %. Of interest, only simultaneous inhibition of JAK + SYK was able to prevent bone and cartilage damage, showing a completely normal joint structure in 60 % of mice. Therefore, concurrent JAK + SYK inhibition ameliorates clinical, immunological and histological parameters of arthritis.

### Dual JAK + SYK inhibition is more effective than anti-TNF treatment

**Fig. 3:**
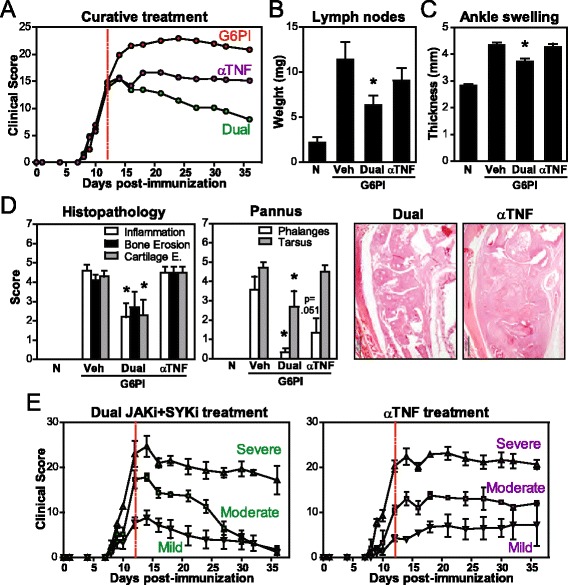
Therapeutic comparison of Janus kinase (*JAK*) + spleen tyrosine kinase (*SYK*) inhibition and anti-TNF therapy in glucose-6-phosphate isomerase (*G6PI*)-induced arthritis. DBA/1 mice were immunized with G6PI + complete Freund’s adjuvant on day 0, after T regulatory cell depletion with anti-CD25 Ab (days −8 and −11). Treatments were administered starting on day 12 (*red dotted line*). Samples were obtained on day 36. **a** Time course for arthritis scores. **b** Mean weight of both inguinal lymph nodes. **c** Mean ankle thickness of both hind paws measured with a manual caliper. **d** Histopathological scores for tarsus and pannus formation in both phalanges and tarsus. Representative histological sections of tarsal joints are also shown (*bar* 500 μM). **e** Time course for arthritis scores for mice with mild, moderate and severe arthritis at the time of treatment initiation. Graphs show mean (± standard error of the mean) for 2–10 mice/group, *p <0.05 versus vehicle control. *N* naive, *Veh* vehicle

We have showed greater efficacy of dual versus single JAK and SYK inhibition in a chronic model of arthritis. Next, we compared the efficacy of dual JAKi + SYKi with anti-TNF therapy, a standard anti-rheumatic biological approach used in the clinics. Previous reports showed that anti-TNF prevents the development of very early acute G6PI arthritis [20, 26]. Here, we started treatment of Treg-depleted G6PI immunized mice when arthritic symptoms were moderate to severe. Interestingly, both TNF blockade (10 mg/kg, every third day) and dual JAKi + SYKi stopped disease worsening but only dual treatment was able to progressively decrease the clinical score, which had decreased by over 60 % at the endpoint (Fig. 3a). The average area under the clinical score curve decreased by 42 % with dual treatment, while it only decreased by 25 % with etanercept (not shown). Accordingly, lymph node weight (Fig. 3b) and ankle thickness (Fig. 3c) were significantly lower in all mice treated with JAKi + SYKi but not with anti-TNF. Moreover, 66 % of dual-treated mice experienced complete recovery of phalangeal joints after 3 weeks of treatment (not shown). Blinded histologic observations also confirmed reduced recruitment of immune and inflammatory cells and joint damage in tarsal joints of mice treated with JAKi + SYKi but not in anti-TNF-treated mice (Fig. 3d). Abnormal layers of granulation and fibrovascular tissue (pannus) were abundant in joints of vehicle-treated mice, which coincides with chronic and severe stages of arthritis. Dual inhibition significantly reduced pannus progression in both tarsal and phalangeal joints, while anti-TNF treatment only had a partial effect in phalanges. Furthermore, Fig. 3e shows that dual kinase inhibition reversed the pathological changes in mice with mild and moderate disease and decreased symptoms in mice with very severe disease. In contrast, blockade of TNF stopped, without improving, disease progression in all groups. Thus, dual JAK + SYK inhibition also presents greater efficacy than anti-TNF treatment in this experimental model of severe arthritis.

### Evaluation of potential immunotoxicity of dual JAK + SYK inhibition

**Fig. 4:**
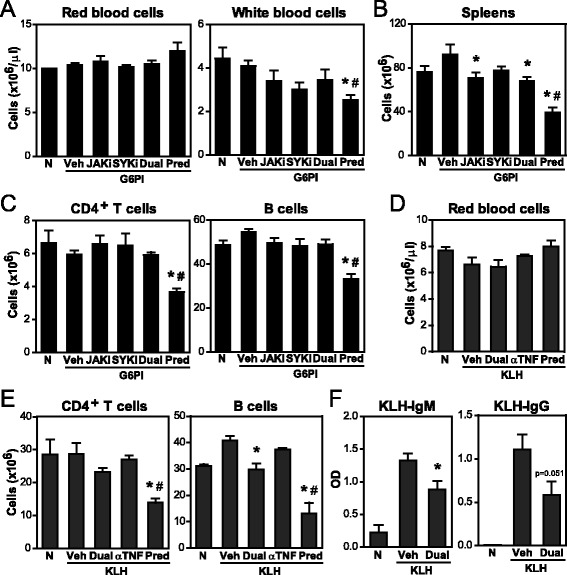
Evaluation of potential immunotoxicity in in vivo assays. **a**-**c** DBA/1 mice were immunized with glucose-6-phosphate isomerase (*G6PI*) + complete Freund’s adjuvant and exposed to treatment daily for 31 days. **a** Red blood cell counts and leucocyte counts in peripheral blood. **b** Splenocyte numbers. **c** Numbers of CD4^+^ T cells (CD3^+^CD4^+^CD8^−^) and B cells (CD3^−^CD19^+^MHCII^+^) in spleens. **d**-**f** CD-1 mice were treated from day −14 to 14 and immunized with keyhole limpet hemocyanin (*KLH*) on day 0 (T cell-dependent antibody response (TDAR) assay). **d** Red blood cell counts in peripheral blood. **e** Numbers of CD4^+^ T cells (CD3^+^CD4^+^CD8^−^) and B cells (CD3^−^CD19^+^MHCII^+^) in the spleen. **f** Levels of KLH-specific IgM and IgGs in plasma at day 7 and 14, respectively. Graphs show mean (+ standard error of the mean) for 3–6 mice/group, *p <0.05 versus vehicle control, ^#^ *p* <0.05 versus non-immunized and untreated group (naive). *N* naive, *Veh* vehicle, *JAKi* Janus kinase inhibitor, *SYKi* spleen tyrosine kinase inhibitor, *Pred* prednisolone

**Fig. 5:**
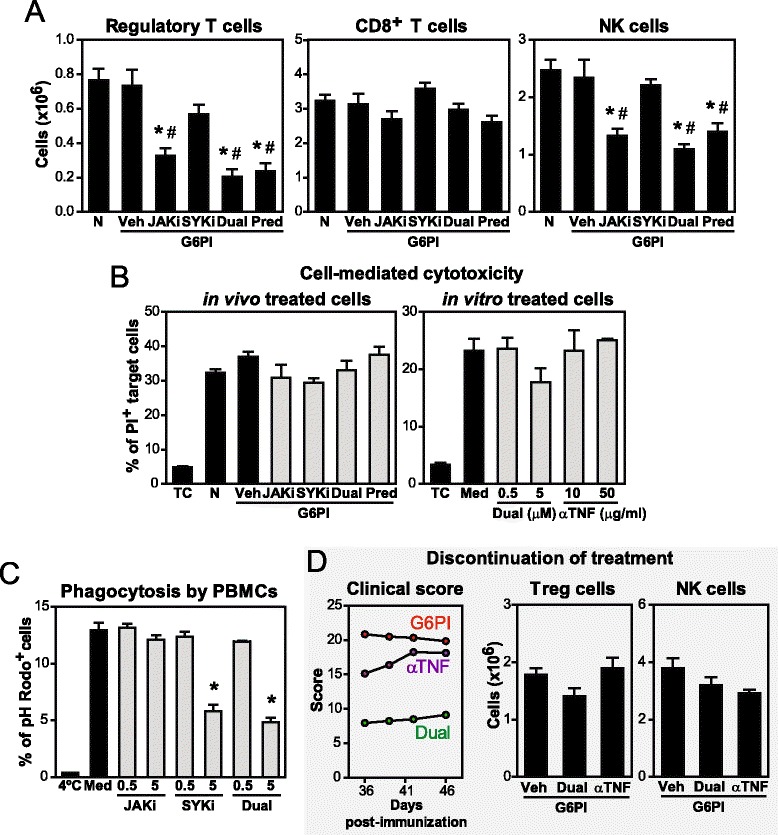
Evaluation of potential immunotoxicity in in vitro*/*in vivo assays and effects of treatment withdrawal. **a** Numbers of T regulatory (*Treg*) cells (CD3^+^CD4^+^CD25^hi^Foxp3^+^), CD8^+^ T cells (CD3^+^CD8^+^CD4^−^) and natural killer (*NK*) cells (CD3^−^CD49b^hi^) in DBA/1 mice immunized with glucose-6-phosphate isomerase (*G6PI*) + complete Freund’s adjuvant and exposed to treatment daily for 31 days. **b** Cell-mediated cytotoxicity assay with splenocytes either treated in vivo (*left*) or in vitro (*right*) and YAC-1 as target cells. **c** Phagocytic activity of peripheral blood mononuclear cells (*PBMC*) treated in vitro. **d** Time course for arthritis scores and numbers of Treg cells and NK cells in the spleen of G6PI-immunized mice treated for 35 days and sacrificed on day 47. Graphs show mean (+ standard error of the mean) for 2–6 mice/group, *p <0.05 versus vehicle or media control, ^#^ *p* <0.05 versus non-immunized and untreated group (naive). *N* naive, *Veh* vehicle, *Predni* prednisolone, *TC* target cells, *Med* media, *JAKi* Janus kinase inhibitor, *SYKi* spleen tyrosine kinase inhibitor

We next evaluated whether the benefit of greater efficacy of dual JAKi + SYKi in arthritis was limited by increased adverse effects imputable to target inhibition. To this end, mouse weight as well as hematological and immunological parameters were examined in mice immunized either with G6PI + CFA (Figs. 4a-c and 5a) or with KLH (Fig. 4d-f) and treated for extended periods of time, i.e., 3 or 4 weeks, respectively. KLH is an immunogen routinely used in the rodent TDAR model, which allows detection of potential immunotoxicity, especially immunosuppression. As shown in Additional file 3a and b, the body weight from mice treated with JAKi/SYKi remained comparable to vehicle-treated animals. As expected, the control immunosuppressant drug prednisolone significantly reduced body weight, circulating white blood cells, spleen weight and both CD4^+^ T cell and B cell counts in G6PI- and KLH-immunized mice. Kinase inhibitors alone or in combination and anti-TNF treatment did not alter red blood cell or white blood cell numbers. Dual JAKi + SYKi and single JAKi decreased spleen weight compared to G6PI-immunized controls but never below naive levels, and without affecting CD4^+^ T cell and B cell counts. In the TDAR model, dual JAK + SYK inhibition, but not etanercept, decreased B cell counts but never below baseline, and consequently, KLH-specific antibody production was also only mildly reduced.

Preclinical studies and clinical trials with tofacitinib have highlighted an impact on Treg cells. Here, we also observed a significant decrease in Treg counts in the spleen of mice treated with tofacitinib and, consequently, also with dual JAKi + SYKi, but not SYKi alone (Fig. 5a). Similar diminished Treg numbers were identified in prednisolone-treated mice.

We also examined potential effects of JAKi, SYKi and TNF blockade on cytotoxic cell populations (Fig. 5a). CD8^+^ T cell counts were similar in all groups. In contrast, dual inhibition, but not anti-TNF (not shown) significantly impacted on natural killer (NK) cell numbers, likely due to a direct effect of JAK inhibition on IL-15 signaling. Under these conditions, we observed a fully functional cell-mediated cytotoxic response of in vivo treated splenocytes on target YAC-1 cells upon co-culture (Fig. 5b left). Comparable results were obtained in another cytotoxic assay with murine splenocytes from naive mice but, this time, treated in vitro with physiologically relevant (0.5 μM), and ten times higher (5 μM), concentrations (Fig. 5b right). Therefore, reduced NK cell numbers upon dual inhibition did not impact overall cytotoxic activity. In a separate experiment, we confirmed that tofacitinib and PRT062607 were not cytotoxic at the assayed doses (Additional file 3C).

In terms of potential effects on the phagocytic cell compartment, human PBMCs showed intact phagocytosis of *Escherichia coli* bioparticles when exposed to 0.5 μM of JAKi and/or SYKi for 1 h (Fig. 5c). At the high concentration of 5 μM, dual inhibition decreased almost 60 % the phagocytic activity of PBMCs, which can be explained by the role of SYK on actin cytoskeleton reorganization during phagocytic vesicle closure. The flow cytometric analysis revealed CD14^+^ cells and granulocytes to be the main phagocytes involved. Again, these adverse effects have a limited impact in vivo, as high plasmatic concentrations are reached only for short periods of time. Overall, our data show that dual JAK + SYK inhibition is efficacious and has a good safety profile, and therefore can be seen as a candidate treatment strategy in arthritis.

### Sustained efficacy and reversed adverse effects after discontinuation of dual treatment

We next stopped treatment and ten days later 75 % of arthritic mice treated with both inhibitors either maintained or continued to ameliorate clinical signs (Fig. 5d). In sharp contrast, discontinuation of anti-TNF treatment led to a worsening of paw inflammation in all mice. Indeed, an average 17 % increase in the clinical score was observed in this group, reaching a score comparable to vehicle-treated arthritic mice. Therefore, the benefits of dual JAK + SYK inhibition were perpetuated longer than anti-TNF. Therapy discontinuation also led to immune recovery. The reduced Treg and NK cell counts after daily JAKi + SYKi were quickly restored to normal levels (Fig. 5e), which minimizes potential adverse effects.

### Dual JAK + SYK inhibition impacts the development and function of synoviocytes and osteoclasts

**Fig. 6:**
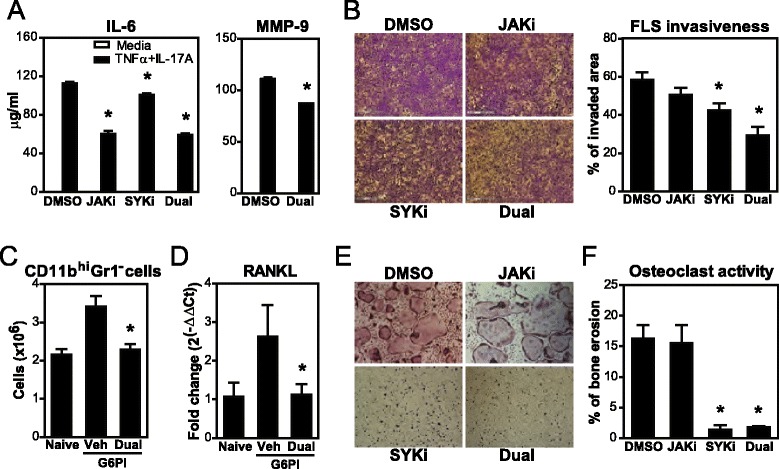
Effects of Janus kinase (*JAK*) + spleen tyrosine kinase (*SYK*) inhibition on fibroblast-like synoviocytes and osteoclasts. **a** Cytokine and matrix metalloproteinase (*MMP*) production by fibroblast-like synoviocytes (*FLS*) from arthritic mice stimulated with 10 ng/ml rmTNF and 50 ng/ml rmIL-17A and treated in vitro with kinase inhibitors (0.5 μM) for 24 h. **b** Representative images after crystal violet staining and data showing the invasive potential of FLS from arthritic mice treated with kinase inhibitors (0.5 μM) in transwell collagen-coated chambers for 48 h. **c** Numbers of CD11b^hi^Gr1^−^cells, which contain most osteoclast precursors, and **d** rank ligand (*RANKL*) expression in spleens of DBA/1 mice immunized with glucose-6-phosphate isomerase + complete Freund’s adjuvant and exposed to JAK inhibitor (*JAKi*) + SYK inhibitor (*SYKi*) daily for a month. **e** Representative images after tartrate-resistant alkaline phosphatase staining showing the impact of JAKi/SYKi on osteoclast differentiation from bone marrow cells after 5 days in a RANKL + M-CSF enriched culture. **f** Activity of mature osteoclasts on cortical bone slices treated with kinase inhibitors (0.5 μM) for 48 h. Graphs show mean (+ standard error of the mean) of a representative experiment of 2–4 with 3–7 samples/group, *p <0.05 versus dimethyl sulfoxide (*DMSO*) or naive control. *Veh* vehicle

Resident cells of the joint are major contributors to arthritis pathology. In particular, FLS and osteoclasts represent a link between joint inflammation and structural damage, which is critical in the functional disability of RA patients. Here, we studied the importance of the JAK and SYK pathways on their function in order to understand the mechanism leading to increased efficacy of dual JAKi + SYKi. FLS were isolated from small joints of mice with chronic arthritis (Treg-depleted). Stimulation of FLS with TNF and IL-17A, which are elevated in arthritic mice (Fig. 1 and Additional file 2) and in the synovium of RA patients [27, 28], induced IL-6 and MMP production (Fig. 6a). Dual JAKi + SYKi markedly reduced IL-6 secretion to a similar extent to JAKi. Milder effects were observed in MMP9 and MMP3 levels (not shown). In a transwell system with collagen-coated inserts, JAKi, SYKi and dual JAKi + SYKi decreased the invasive capacity of FLS by 13, 27 and 50 %, respectively (Fig. 6b). Therefore, JAK mediated cytokine production, and both JAK and SYK additively contributed to collagen invasiveness, a critical step in cartilage destruction.

In bone destruction, osteoclasts are the primary resorbing cells. Here, we found that G6PI-immunized mice treated with JAKi + SYKi had baseline counts of osteoclast precursors (CD11b^hi^Gr1^−^ cells) and levels of RANKL expression in spleens (Fig. 6c and d). Furthermore, in vitro osteoclastogenesis from bone marrow cells was completely prevented with SYKi and dual JAKi + SYKi exposure (Fig. 6e). We also exposed mature osteoclasts to these inhibitors and observed that SYK, but not JAK, is critically required for bone resorption activity (Fig. 6f). Thus, dual JAKi + SYKi treatment interferes at multiple stages in osteoclast development and function, primarily via SYK.

### Dual JAK + SYK inhibition also regulates effector and memory CD4^+^T cells and plasma cells

**Fig. 7:**
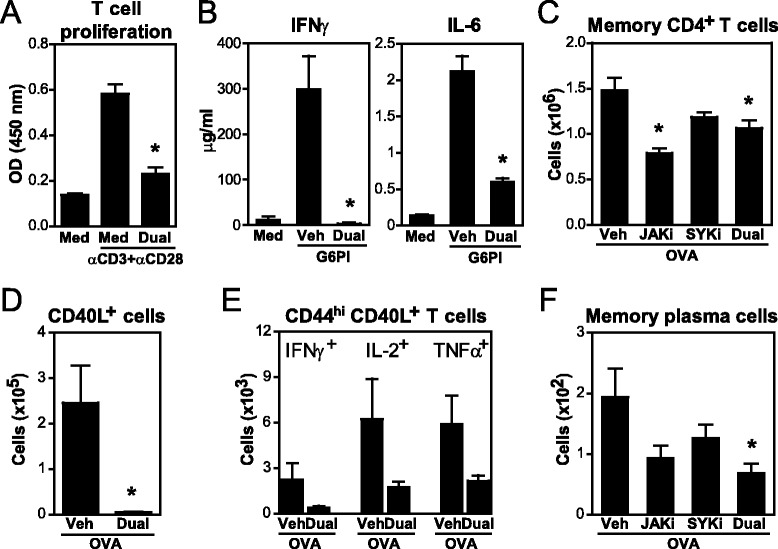
Effects of Janus kinase (JAK) + spleen tyrosine kinase (SYK) inhibition on effector and memory T and B cells. **a** Proliferation of purified T cells stimulated with 1 μg/ml anti-CD3 and 0.5 μg/ml anti-CD28 Abs in the presence of kinase inhibitors (0.5 μM) for 72 h. **b** Cytokine secretion by splenocytes from arthritic mice in vitro re-exposed to 20 μg/ml recombinant human glucose-6-phosphate isomerase (G6PI) and treated with JAK inhibitor (*JAK*i) + SYK inhibitor (*SYKi*) (0.5 μM) for 72 h. **c**-**f** BALB/c mice were immunized and challenged with ovalbumin (*OVA*) + alum; 2 weeks after challenge, mice were treated with kinase inhibitors for 11 days. **c**-**e** Numbers of memory CD4^+^ T cells (CD3^+^CD4^+^CD44^hi^), antigen-specific (CD3^+^CD4^+^CD44^hi^CD40L^+^) and cytokine-producing (CD40L^+^cytokine^+^) memory CD4^+^ T cells upon OVA re-exposure in vitro. **f** Numbers of OVA-specific memory plasma cells (CD138^+^κλ^hi^OVA^+^ 5-Bromo-2′-deoxyuridine^+^). Graphs show mean (+ standard error of the mean) for 3–5 mice/group, *p <0.05 versus media or vehicle control. *Med* media, *Veh* vehicle

The JAK/STAT pathway is known to mediate T cell differentiation by interfering with cytokine signaling. Our data show that dual JAKi + SYKi prevented T cell proliferation upon unspecific stimulation in vitro (Fig. 7a). Antigen-specific responses, primarily IFNγ and IL-6 production, were also markedly decreased in splenocytes from arthritic mice treated in vitro with both inhibitors (Fig. 7b).

Cells of the immunological memory, in particular memory T cells and long-lived plasma cells are implicated in the chronification of disease and have proven to be refractory to conventional immunosuppression [29]. In order to investigate the role of JAK and SYK on memory cell maintenance, OVA-immunized mice were treated with inhibitors starting 2 weeks after challenge, a time when memory cells are already generated. The number of memory CD4^+^CD44^hi^ T cells was similarly decreased in mice treated with JAKi and dual JAKi + SYKi (Fig. 7c). Upon OVA re-exposure in vitro, JAKi + SYKi markedly reduced antigen-specific (CD40L^+^) CD4^+^ T cells and their ability to produce IFNγ, IL-2 and TNFα (Fig. 7d and e). OVA-specific memory plasma cells (CD138^+^κλ^hi^) were also significantly reduced by an additive effect on JAK and SYK signaling (Fig. 7f). Thus, both JAK and SYK mediate memory cell pool maintenance and, thus, their inhibition could maintain disease remission and prevent arthritis recurrence.

## Discussion

Tofacitinib has demonstrated high efficacy in phase II and III trials and even in patients not responding to current treatments. However, the response is limited (ACR20 48–51 %) [12, 13], so there are still difficult-to-treat patients with RA in need of new therapeutic approaches. To our knowledge, no clinical trial has evaluated the potential benefits of a selective SYK inhibitor in RA patients. Here, we studied whether the addition of a SYK inhibitor into a JAK inhibition therapy could improve the efficacy achieved by single inhibition. We used the murine Treg-depleted G6PI-induced arthritis model, a clinically relevant model characterized by non-remitting and progressive peripheral polyarthritis with loss of joint structure and function [21].

In a preventive protocol, our data clearly show that combined JAKi + SYKi offers greater prophylactic efficacy than single kinase inhibition (Fig. 1). Mice treated with JAKi alone had very mild disease that completely resolved within 2 weeks. At study termination, there were no signs of inflammation and joint morphology looked normal. These results can be in part explained by the importance of JAK on Th cell differentiation and function [30], and the critical role of CD4^+^ T cells in the induction phase of this chronic G6PI model [21] and others [31]. Here, we found that antigen-specific T cell responses were similarly reduced in JAKi- and JAKi + SYKi-treated mice. Inhibition of JAKs also reduced the number of activated DCs. Recent reports show that JAK inhibition profoundly modulates DC development and activation, which is most likely due to signaling blockade of the critical differentiating signals GM-CSF and IL-4 [32–34]. In this scenario of reduced DC and T cell activation, it is not surprising to see lower numbers of activated B cells and G6PI-specific IgGs upon JAK inhibition. Single inhibition of SYK led to the development of milder arthritis. A greater role for SYK on disease induction has been reported in other models; in particular, genetic deficiency of Syk in the hematopoietic compartment completely blocked the development of arthritis in the K/BxN serum-transfer murine model [35] and Coffey et al. [19] reported PRT062607 to dose-dependently reduce the development of paw inflammation in a murine CAIA model. While greater doses of SYKi could be administered to likely enhance efficacy, we did not want to compromise kinase selectivity. Mice treated with SYKi had decreased numbers of activated DCs, B cells and IgG levels, likely explained by the role of SYK on B cell receptor (BCR) signaling and DC maturation via C type lectin receptors [36]. Thus, both JAK and SYK signaling pathways are involved in early pathogenic mechanisms of arthritis and simultaneous inhibition of both kinases results in greater efficacy.

We also followed a curative protocol (Fig. 2), in which treatment was started at day 12 post-immunization, when clinical signs were clearly present and affected multiple joints. Under these conditions, inhibition of SYK resulted in a minimal (non-significant) decrease of arthritis parameters. Our results are in contrast with a previous preclinical study in the rat CIA model showing significant efficacy of PRT062607 when treatment was initiated early, i.e., when at least one hind paw showed first signs of inflammation (score 1) [19]. These differences in efficacy may result from using distinct experimental models (CIA in rat is acute and less stringent) and starting treatment at different disease stages (mild versus moderate). Inhibition of JAK immediately stopped disease worsening and started to ameliorate clinical signs after 2 weeks of daily treatment, ending with moderate disease. These results parallel the responses of arthritic patients to tofacitinib, i.e., significant clinical and physical improvement as early as 2 weeks and inhibition of progress in joint destruction after 6 months of treatment [9, 12, 14]. This lower efficacy of JAKi in the curative protocol compared to the preventive one can be partially explained by the reduced importance of effector CD4^+^ T cells when disease is already established. Indeed, Frey et al. [21] reported that the effector phase of arthritis in the Treg-depleted G6PI model is non-dependent on CD4^+^ T cells, when other cell populations, such as phagocytes, are more relevant. If multiple local cells of the joint, rather than adaptive immune cells, drive disease progression during the effector phase of arthritis, it is not surprising to see that therapeutically targeting two signaling pathways is more effective than targeting only one. Indeed, the observed changes after dual JAKi + SYKi were impressive, with no symptoms to minimal symptoms at study termination. In fact, dual inhibition decreased clinical scores in all mice with distinct degrees of arthritis severity, showing 87 %, 90 % and 30 % reduction in mild, moderate and severe arthritis, respectively (Fig. 3d).

To further support this notion of broader amelioration with multi-pathway inhibition, dual JAKi + SYKi also resulted in higher efficacy than anti-TNF therapy, the most common first choice biologic strategy (ACR guidelines 2012) (Fig. 3). Blockade of TNF with etanercept stopped, without improving, disease progression in mice with mild, moderate and severe arthritis, resulting in an average clinical score 25 % lower than vehicle-treated mice, but 39 % higher than dual treatment. Furthermore, partial arthritis amelioration after etanercept treatment was only observed in phalangeal joints, as tarsus remained with maximal inflammatory infiltrates, pannus and severe bone/cartilage erosion. To confirm the activity of the drug used, we found that etanercept was able to prevent disease onset in CIA mice (not shown). Thus, targeting only one pro-inflammatory signal when severe and erosive disease is already established results in reduced and slower recovery than dual JAKi + SYKi treatment. The observed partial efficacy of TNF blockade under these severe conditions is not surprising, as 30–40 % of RA patients have an inadequate response to TNF inhibitors [37–39]. The increased efficacy of our proposed mechanism of action in this demanding model of arthritis, in which other therapies fail, suggests JAKi + SYKi as a potential pharmacological tool to treat patients not responding to current treatments. Our data also endorse the use of the chronic G6PI model to investigate difficult-to-treat arthritis.

The benefits of dual JAKi + SYKi need to be considered in the context of the risks of adverse events. The most common target-related adverse effects observed in clinical trials with JAK include a higher incidence of infection (resulting from immunosuppression) and anemia [14]. Of note, companies have minimized or overcome some adverse events by optimizing the dose and posology. Our data revealed that a month of daily treatment with JAKi + SYKi did not reduce total circulating leucocytes or red blood cell counts, hemoglobin or hematocrit levels in arthritic (Fig. 4) and healthy KLH-immunized mice (not shown). The weight and cellularity (not shown) of spleens and lymph nodes were decreased but never below those from naive mice or those treated with prednisolone, a well-known immunosuppressive agent. A phenotypic analysis in the spleen revealed no significant changes on CD4^+^ T or B cell counts in all treated arthritic mice, albeit B cells were reduced to baseline numbers in KLH-immunized mice. Such reduction was paralleled by lower KLH-specific IgG production, which is consistent with the observed reduction in the levels of G6PI-specific IgG (Fig. 1). Importantly for host defense against pathogens, the ability to generate antibodies was never completely abolished by single or dual treatment, allowing these treated mice to still mount robust antigen-specific humoral responses. A study in monkeys also reported none to modest effects on leucocytes, T and B cell counts after chronic (3-week), oral tofacitinib administration [40]. In contrast, mice deficient on JAK3 (JAK3^−/−^), common γc (γc^−/−^) or receiving tofacitinib by infusion with osmotic minipumps showed marked reductions in T and B cell numbers [41–44]. These suggest that intermittent, rather than continuous, kinase inhibition reduces immunosuppression without compromising efficacy.

Non-specific defense mechanisms, including cytotoxicity and phagocytosis, were also preserved in mice exposed to physiologically relevant concentrations of JAKi + SYKi (Fig. 5). Numbers of CD8^+^ T cells and neutrophils (not shown) were comparable among healthy and treated mice. In contrast, NK cell counts significantly decreased 43 % and 53 % after chronic JAKi or JAKi + SYKi, respectively. This selective effect on NK cells was already observed after 6 days of treatment (not shown) and is in accordance with the reported time- and dose-dependent reduction of circulating NK cells after tofacitinib administration [40]. JAK3^−/−^ and γc^−/−^ mice also exhibited a profound loss of NK cells [44], which is consistent with the known role of IL-15 and IL-21 for their homeostasis [45]. Importantly, a reduction only in this population was not sufficient to impact overall cytotoxicity. After chronic treatment with dual JAKi + SYKi, the number of CD3^+^CD4^+^CD25^bright^Foxp3^+^ T cells (Treg) decreased 70 % from baseline. A comparable decrease was observed in mice treated with JAKi, which could be explained by the critical requirement of IL-2/JAK1&3/STAT5 signaling for the maturation of FOXP3^+^ Tregs [46, 47]. Decreased Treg counts after chronic tofacitinib administration has been reported by others that further demonstrate that the regulatory capacities of the residual Treg cells remain normal [48]. Collectively, our data show that the potential target-related adverse effects of dual inhibition do not increase compared to each single inhibition and are mitigated because we managed to transiently inhibit JAK and SYK signaling. In particular, 6–12 h compound coverage (Additional file 1) was sufficient to ameliorate the disease in this model and allow enough time for most basic physiological processes to recover homeostasis.

It is important for safety and economic reasons to consider the possibility of therapy withdrawal. Here we show that the significant reduction in Treg and NK cell counts in mice chronically exposed to JAKi + SYKi was quickly reversed after treatment discontinuation (Fig. 5). Interestingly, the clinical score of these mice remained low, suggesting long-lasting effects of dual treatment. In sharp contrast, discontinuation of anti-TNF treatment led to a prompt rebound of disease, i.e., clinical scores reached arthritic control severity in less than a week. Thus, the specific immune suppression resulting from dual JAKi + SYKi treatment can be easily reversed without compromising efficacy. Further studies should determine the optimal treatment regimen to preserve the clinical benefit, avoid disease recurrence and reduce adverse events.

In order to understand the mechanism leading to increased efficacy by dual JAKi + SYKi, we studied the contribution of these kinase pathways on specific cell subsets relevant to disease pathology (Figs. 6 and 7). Local FLS and osteoclasts are major effectors of joint inflammation (synovitis) and destruction, and therefore are important therapeutic targets in RA. Our data show that JAKi + SYKi significantly reduced the aggressive phenotype of arthritic FLS (Fig. 6a and b). Interestingly, cytokine production upon TNF + IL-17A stimulation was primarily JAK-dependent, while SYK signaling contributed more to FLS invasiveness. In this regard, the JAK-STAT pathway has been implicated in TNF and IL-17 signaling via autocrine production of IFNβ and PI3K activation, respectively [49–51]; and SYK is involved in the signaling of integrins that mediate FLS adhesion and invasion to the extracellular matrix, an important initiating step in the progressive destruction of articular cartilage (reviewed in [52, 53]). Dual JAKi + SYKi also prevented extensive bone destruction by altering both osteoclastogenesis and osteoclast activity (Fig. 6c-f). In particular, we found that mice treated with JAKi + SYKi had fewer CD11b^hi^Gr1^-/lo^ splenocytes, a population with osteoclast-forming potential; in fact, 60–70 % of these cells are known to differentiate into TRAP^+^ osteoclasts when cultured with RANKL and M-CSF [54, 55]. Dual JAKi + SYKi, solely via SYKi, completely blocked intercellular fusion and bone erosion activity but osteoclasts still showed partial TRAP activity (not shown). This is in accordance with syk^−/−^ osteoclasts, which do not differentiate morphologically but still express mature osteoclast markers, and also show impaired functional resorption of mineralized matrix [18]. These alterations are likely explained by the importance of SYK on cytoskeletal rearrangements and specific integrin-mediated functions (reviewed in [56, 57]. These in vitro results along with our histopathological data from arthritic mice (Fig. 2g) reinforce the idea that affecting multiple cell types with dual inhibition is more effective than single inhibition in this complex system.

There are a growing number of reports pointing to the importance of chronically activated synovial T cells for the stimulation of resident FLS and osteoclast precursors. Our data show that dual JAKi + SYKi markedly reduced T cell proliferation and cytokine production upon unspecific stimulation or antigen re-exposure, suggesting a counteracting effect on aberrant T cell activity (Fig. 7). It is likely that this effect was IL-2 driven, and, thus, more dependent on JAK. RANKL expression, primarily found on T cells but also B cells and FLS [58, 59], was also significantly lower in JAKi + SYKi-treated mice, which further contributes to decreasing osteoclast differentiation and activity. T and B cells have been suggested to also play a key role in the perpetuation and recurrence of symptoms, in part due to the generation of immunological memory. Therefore, combating autoimmune memory, yet a challenge, is seen as an important clinical goal. Here, our data show that the memory CD4^+^ T cell pool and, to a greater extent, the antigen-specific effector subset were significantly reduced after JAKi + SYKi treatment. As treatment was started 2 weeks after antigen challenge, a time when memory cells are already generated [60, 61], our data suggest an effect of JAKi on memory T cell maintenance. In this regard, IL-7 is known to be critical for memory CD4 cell survival [62], and IL-7 signals via the JAK1/JAK3-STAT5 pathway. Dual treatment also reduced the number of antigen-specific memory plasma cells (CD138^+^κλ^hi^). While the factors involved in plasma cell survival are poorly understood, we speculate that blockade of IL-6 signaling via the JAK1-STAT3 pathway, and perhaps B cell activating factor (BAFF) responsiveness, which has recently been shown to require SYK, may explain the reduced memory plasma cell counts [63, 64].

RA initiates many years before clinical onset, which raises the demands for prevention and early diagnosis. Our data suggest simultaneous inhibition of JAK + SYK to be a very efficacious treatment strategy at early stages of disease development, as it interferes with multiple steps of the immunization process, i.e., activation of DCs, B cells and T cells. Our work also shows that dual inhibition is more effective than current therapeutic strategies, namely anti-TNF or single JAKi, in ameliorating severe disease manifestations. Paw inflammation, bone erosion and cartilage damage were significantly reduced in mice treated with JAKi + SYKi, as a result of interfering in systemic T/B cell functions and in local destructive events, primarily osteoclast and FLS activity. Thus, efficacy resulted from interfering with the activity of multiple cells, and perhaps reducing compensatory disease mechanisms. Importantly, intermittent suppression of individual targets was sufficient to modulate disease activity and reduce adverse events. Overall, the greater and broader anti-rheumatic action of adding SYK inhibition to a JAK inhibition therapy hold experimental promise but will need confirmation from clinical studies.

## Conclusions

The present study collectively suggests that dual JAK + SYK inhibition with selective, potent and orally bioavailable small molecules could complement the current arsenal of tools in development for the treatment of rheumatoid arthritis and likely other inflammatory and autoimmune disorders.

## Additional files

Additional file 1:
**Potency, selectivity and pharmacokinetics of tofacitinib/CP-690,550 (Janus kinase inhibitor** **(** ***JAKi*** **))** **and PRT-062607 (spleen tyrosine kinase inhibitor (** ***SYKi*** **)).** **a**, **b** Human cell lines were pre-incubated with increasing concentrations of JAKi or SYKi (0.005 to 5 μM) for 1 h and then stimulated with either 100 ng/ml rmIL-6 for 15 minutes or 10 μg/ml anti-IgM for 5 minutes. Cells were fixed, permeabilized, stained with anti-STAT3(pY705)-Alexa Fluor 647 or anti-BLNK(pY84)-Alexa Fluor 488 and quantified by flow cytometry. Half maximal inhibitory concentration (*IC*_*50*_) was extrapolated from an inhibition vs log (concentration) curve. **c** Collagen-induced arthritis (*CIA*) model. DBA/1 mice were immunized on day 0 with type II collagen (100 μg/mouse, subcutaneous administration (s.c.)) emulsified in complete Freund’s adjuvant (CFA) and boosted on day 21. Starting on day 30, when disease was mild, mice were orally treated once a day with increasing doses of JAKi (3.75 to 30 mg/kg). Graph shows mean for 6–8 mice/group. **d** Collagen antibody-induced arthritis (*CAIA*) model. BALB/c mice received a cocktail of monoclonal antibodies (2 mg, intravenous administration (i.v.)) directed against type II collagen on day 0 and a lipolysaccharide challenge (70 μg, intraperitoneal administration (i.p.)) on day 3. Twice daily treatment with 30 mg/kg SYKi started on day 4. Graph shows mean for 6–8 mice/group. For monitoring both CIA and CAIA models, each paw was scored individually on a scale of 0–4, with 4 indicating the most severe swelling and erythema. **e** DBA/1 mice received a single oral dose of JAKi (20 mg/kg) or SYKi (30 mg/kg) and plasmatic drug concentration was determined by liquid chromatography-tandem mass spectrometry. Graph shows mean for 2–7 mice/group and a line representing IC_50_ values obtained in **a** and **b**. (PDF 755 kb) Additional file 2:
**Clinical and immunological comparison of acute versus chronic glucose-6-phosphate isomerase (** ***G6PI*** **)-induced arthritis model in mice.** **a** Diagram summarizing the experimental protocol followed in Figs. 1, 2, 3, 4 and 5 (see “Methods” for more details). **b** Time course for arthritis scores clearly showing moderate and acute arthritis in G6PI-immunized mice, and severe and chronic arthritis in T regulatory cell (*Treg*)-depleted and G6PI-immunized mice. **c** Proportion of CD25^hi^Foxp3^+^ Treg cells in CD3^+^CD4^+^ cells at the time of immunization (day 0). **d** Numbers of total cells from inguinal lymph nodes and **e** leucocyte counts in peripheral blood at day 6 post-immunization. **f** Cytokine secretion by splenocytes isolated on day 31 and stimulated in vitro with 20 μg/ml G6PI for 72 h. Graphs show mean (± standard error of the mean) for 3–6 mice/group, **p <*0.05 versus naive control, ^#^ *p <*0.05 versus G6PI-immunized mice. *s.c.* subcutaneous administration, i.v. intravenous administration, *i.p.* intraperitoneal administration, *p.o.* oral administration, *QD* one dose a day. (PDF 521 kb) Additional file 3:
**Effect of Janus kinase inhibitor (** ***JAKi*** **)/spleen tyrosine kinase inhibitor (** ***SYKi*** **) treatment on body weight and cell viability.** **a** DBA/1 mice were immunized with glucose-6-phosphate isomerase (*G6PI*) + complete Freund’s adjuvant (CFA) on day 0. **b** CD-1 mice were immunized with keyhole limpet hemocyanin (KLH) on day 14 (T cell-dependent antibody response (*TDAR*) assay). **a, b** Relative body weight to day 0 (before treatment started) was recorded regularly. As expected, naive animals progressively gained weight over time and treatment with the glucocorticoid prednisone induced the most significant decrease in body weight. G6PI immunization triggered the development of arthritis and, therefore, vehicle-treated animals initially lost weight and then slowly recovered. Modest changes were observed in vehicle-exposed mice upon KLH immunization. Body weight in mice daily treated with single or dual JAK/SYK inhibition was comparable to, or even better than, vehicle-treated animals, clearly showing that treatment with JAKi + SYKi did not negatively impact the general wellbeing of mice. **c** HepG2 cells were exposed for 72 h to increasing doses of JAKi/SYKi (0.5, 5 or 50 μM) or diclofenac (positive control of hepatotoxicity) and mitochondrial activity was evaluated by Alamar Blue fluorescence. JAKi did not show any cytotoxic effect at the assayed concentrations. SYKi significantly decreased cell viability only at the highest dose (50 μM). Importantly, plasma levels of SYKi peaked at 3–4 μM and for a very short time (see Additional file 1e); therefore, higher and sustained concentrations are never reached in vivo. These data show that combined inhibition of JAK + SYK does not result in higher general toxicity. Graphs show mean (± standard error of the mean) for 3–6 mice/group or 2–10 replicates/condition, **p <*0.05 versus media (untreated cells). *Med* media, *Diclo* diclofenac, *Predni* prednisolone. (PDF 432 kb)

## Abbreviations

ACR20: American College of Rheumatology 20 % improvement
AUC: area under the curve
BrdU: 5-bromo-2-deoxyuridine
BSA: bovine serum albumin
CAIA: collagen antibody-induced arthritis
CDC: cell-mediated cytotoxicity
CFA: complete Freund’s adjuvant
CIA: collagen-induced arthritis
DC: dendritic cell
DMARD: disease modifying anti-rheumatic drug
DMEM: Dulbecco’s modified Eagle’s medium
DMSO: dimethyl sulfoxide
EC: effector cells
ELISA: enzyme-linked immunosorbent assay
FBS: fetal bovine serum
FLS: fibroblast-like synoviocyte
G6PI: glucose-6-phosphate isomerase
GM-CSF: granulocyte macrophage colony-stimulating factor
H&E: hematoxylin and eosin
IC_50_: half maximal inhibitory concentration
IL: interleukin
IFN: interferon
i.p.: intraperitoneal administration
i.v.: intravenous administration
JAK: Janus kinase
JAKi: Janus kinase inhibitor
kDa: kiloDalton
KLH: keyhole limpet hemocyanin
MAPK: mitogen-activated protein kinase
MMP: matrix metalloproteinase
MTX: methotrexate
NK: natural killer cell
NSAID: non-steroidal anti-inflammatory drug
PBS: phosphate-buffered saline
p.o.: oral administration (*per os*)
PBMC: peripheral blood mononuclear cell
OVA: ovalbumin
QD: one dose a day (*quaque die*)
RA: rheumatoid arthritis
RANKL: receptor activator of nuclear factor kappa-B ligand
rhu: recombinant human
s.c.: subcutaneous administration
SYK: spleen tyrosine kinase
SYKi: spleen tyrosine kinase inhibitor
TC: target cells
TDAR: T cell-dependent antibody response
Th: T helper
TNF: tumor necrosis factor
TRAP: tartrate-resistant acid phosphatase
Treg: regulatory T cells
