# Supplementary figures and images for: Simultaneous inhibition of JAK and SYK kinases ameliorates chronic and destructive arthritis in mice
Source: Arthritis Res Ther. 2015 Dec 10;17:356. doi: 10.1186/s13075-015-0866-0 (PMC4675041; doi:10.1186/s13075-015-0866-0)

Figure Additional 1.

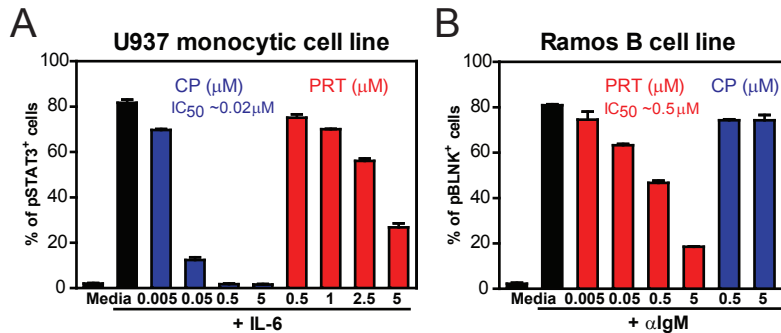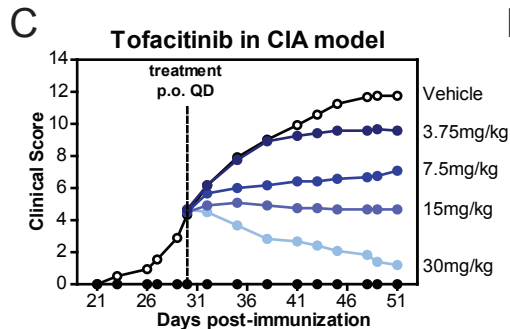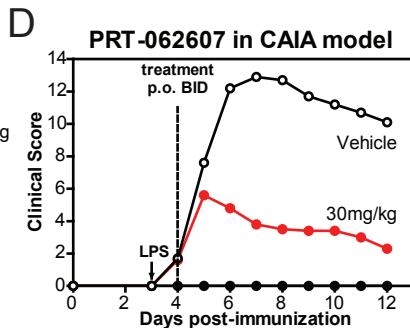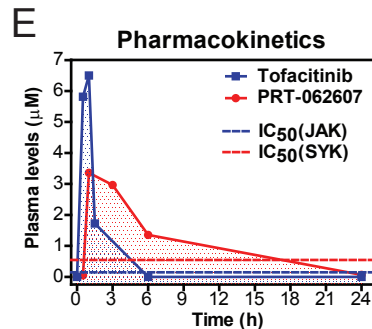

Supplement: Additional file 1: — Potency, selectivity and pharmacokinetics of tofacitinib/CP-690,550 (Janus kinase inhibitor ( JAKi )) and PRT-062607 (spleen tyrosine kinase inhibitor ( SYKi )). a, b Human cell lines were pre-incubated with increasing concentrations of JAKi or SYKi (0.005 to 5 μM) for 1 h and then stimulated with either 100 ng/ml rmIL-6 for 15 minutes or 10 μg/ml anti-IgM for 5 minutes. Cells were fixed, permeabilized, stained with anti-STAT3(pY705)-Alexa Fluor 647 or anti-BLNK(pY84)-Alexa Fluor 488 and quantified by flow cytometry. Half maximal inhibitory concentration (IC 50) was extrapolated from an inhibition vs log (concentration) curve. c Collagen-induced arthritis (CIA) model. DBA/1 mice were immunized on day 0 with type II collagen (100 μg/mouse, subcutaneous administration (s.c.)) emulsified in complete Freund’s adjuvant (CFA) and boosted on day 21. Starting on day 30, when disease was mild, mice were orally treated once a day with increasing doses of JAKi (3.75 to 30 mg/kg). Graph shows mean for 6–8 mice/group. d Collagen antibody-induced arthritis (CAIA) model. BALB/c mice received a cocktail of monoclonal antibodies (2 mg, intravenous administration (i.v.)) directed against type II collagen on day 0 and a lipolysaccharide challenge (70 μg, intraperitoneal administration (i.p.)) on day 3. Twice daily treatment with 30 mg/kg SYKi started on day 4. Graph shows mean for 6–8 mice/group. For monitoring both CIA and CAIA models, each paw was scored individually on a scale of 0–4, with 4 indicating the most severe swelling and erythema. e DBA/1 mice received a single oral dose of JAKi (20 mg/kg) or SYKi (30 mg/kg) and plasmatic drug concentration was determined by liquid chromatography-tandem mass spectrometry. Graph shows mean for 2–7 mice/group and a line representing IC50 values obtained in a and b. (PDF 755 kb) [file 13075_2015_866_MOESM1_ESM.pdf]

Figure Additional 2.

A

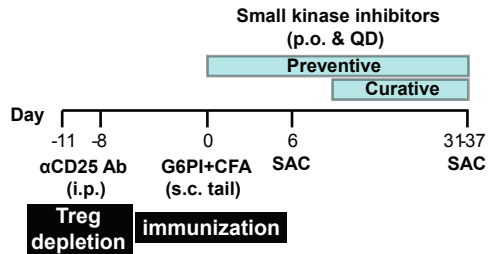

B

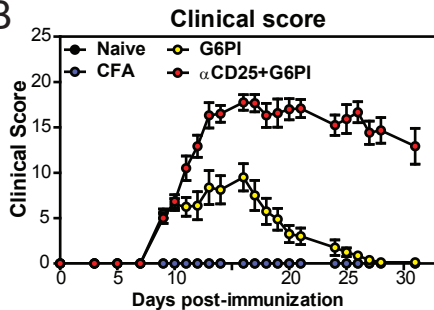

C

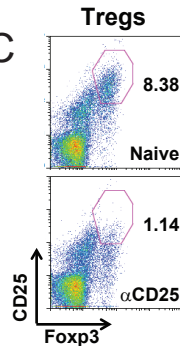

D

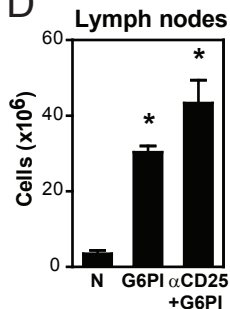

E

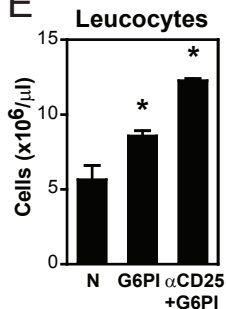

F

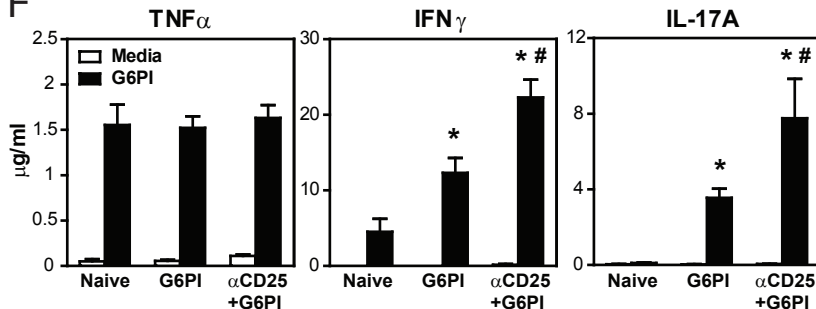

Supplement: Additional file 2: — Clinical and immunological comparison of acute versus chronic glucose-6-phosphate isomerase ( G6PI )-induced arthritis model in mice. a Diagram summarizing the experimental protocol followed in Figs. 1, 2, 3, 4 and 5 (see “Methods” for more details). b Time course for arthritis scores clearly showing moderate and acute arthritis in G6PI-immunized mice, and severe and chronic arthritis in T regulatory cell (Treg)-depleted and G6PI-immunized mice. c Proportion of CD25hiFoxp3+ Treg cells in CD3+CD4+ cells at the time of immunization (day 0). d Numbers of total cells from inguinal lymph nodes and e leucocyte counts in peripheral blood at day 6 post-immunization. f Cytokine secretion by splenocytes isolated on day 31 and stimulated in vitro with 20 μg/ml G6PI for 72 h. Graphs show mean (± standard error of the mean) for 3–6 mice/group, *p <0.05 versus naive control, # p <0.05 versus G6PI-immunized mice. s.c. subcutaneous administration, i.v. intravenous administration, i.p. intraperitoneal administration, p.o. oral administration, QD one dose a day. (PDF 521 kb) [file 13075_2015_866_MOESM2_ESM.pdf]

Figure Additional 3.

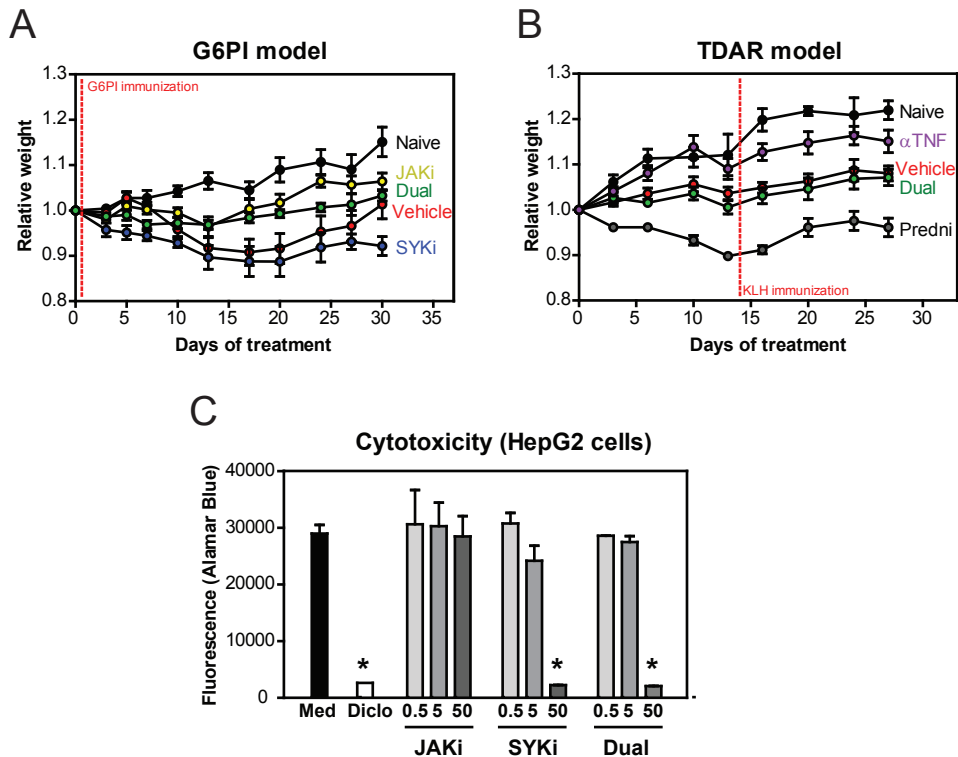

Supplement: Additional file 3: — Effect of Janus kinase inhibitor ( JAKi )/spleen tyrosine kinase inhibitor ( SYKi ) treatment on body weight and cell viability. a DBA/1 mice were immunized with glucose-6-phosphate isomerase (G6PI) + complete Freund’s adjuvant (CFA) on day 0. b CD-1 mice were immunized with keyhole limpet hemocyanin (KLH) on day 14 (T cell-dependent antibody response (TDAR) assay). a, b Relative body weight to day 0 (before treatment started) was recorded regularly. As expected, naive animals progressively gained weight over time and treatment with the glucocorticoid prednisone induced the most significant decrease in body weight. G6PI immunization triggered the development of arthritis and, therefore, vehicle-treated animals initially lost weight and then slowly recovered. Modest changes were observed in vehicle-exposed mice upon KLH immunization. Body weight in mice daily treated with single or dual JAK/SYK inhibition was comparable to, or even better than, vehicle-treated animals, clearly showing that treatment with JAKi + SYKi did not negatively impact the general wellbeing of mice. c HepG2 cells were exposed for 72 h to increasing doses of JAKi/SYKi (0.5, 5 or 50 μM) or diclofenac (positive control of hepatotoxicity) and mitochondrial activity was evaluated by Alamar Blue fluorescence. JAKi did not show any cytotoxic effect at the assayed concentrations. SYKi significantly decreased cell viability only at the highest dose (50 μM). Importantly, plasma levels of SYKi peaked at 3–4 μM and for a very short time (see Additional file 1e); therefore, higher and sustained concentrations are never reached in vivo. These data show that combined inhibition of JAK + SYK does not result in higher general toxicity. Graphs show mean (± standard error of the mean) for 3–6 mice/group or 2–10 replicates/condition, *p <0.05 versus media (untreated cells). Med media, Diclo diclofenac, Predni prednisolone. (PDF 432 kb) [file 13075_2015_866_MOESM3_ESM.pdf]
